# Production of ^64^Cu and ^67^Cu with accelerator neutrons by deuterons and their separation from zinc

**DOI:** 10.3389/fnume.2025.1657125

**Published:** 2025-10-09

**Authors:** Yasuki Nagai, Masako Kawabata, Hideya Saeki, Shoji Motoishi, Kazuyuki Hashimoto, Kazuaki Tsukada, Arata Motomura, Akio Ohta, Naoki Takashima, Shintaro Hashimoto, Masatoshi Itoh, Hidetoshi Kikunaga, Shozo Furumoto

**Affiliations:** ^1^ Oarai Research Center, Chiyoda Technol Corporation, Oarai, Japan; ^2^ Research Center for Accelerator and Radioisotope Science, Tohoku University, Sendai, Japan; ^3^ Research Center for Nuclear Physics, Osaka University, Osaka, Japan; ^4^ Radiation Source Production Section, Chiyoda Technol Corporation, Tokai, Japan; ^5^ Department of Research Infrastructure Technology Development, Japan Atomic Energy Agency, Tokai, Japan; ^6^ Advanced Science Research Center, Japan Atomic Energy Agency, Tokai, Japan; ^7^ Nuclear Science and Engineering Center, Japan Atomic Energy Agency, Tokai, Japan; ^8^ Research Center for Accelerator and Radioisotope Science, Tohoku University, Sendai, Japan

**Keywords:** Copper−64, Copper−67, theranostics, accelerator neutron, deuteron accelerator

## Abstract

The ^64^Cu/^67^Cu pair is an ideal set of theranostic radionuclides for treating patients based on their genetic profiles. We propose a novel production route for this radionuclide pair using accelerator-generated neutrons. We report experimental measurements of the absolute activity and radionuclide purity of ^64^Cu and ^67^Cu, produced by irradiating ^64^Zn and ^68^Zn with these neutrons. The measured results were consistent with simulated values. ^64^Cu and ^67^Cu were separated from the irradiated natural Zn and ^68^Zn using sublimation and column separation techniques. The production methods for ^64^Cu and ^67^Cu developed in this study are expected to enhance their availability in an economically sustainable manner.

## 1 Introduction

Recently, there has been increased interest in personalized nuclear medicine. Currently, a variety of radiopharmaceuticals are administered to cancer patients for therapeutic and diagnostic purposes (1–3). These radiopharmaceuticals contain radionuclides with similar chemical and biochemical properties that allow them to target specific diseases. The term “theranostic” refers to the combined use of diagnostic and therapeutic agents containing these radionuclides (4).

When radioisotopes are used to treat cancer patients, real-time imaging is performed using pharmaceutical compounds labeled with gamma-ray-emitting diagnostic radioisotopes. This allows the distribution of pharmaceutical compounds in the patient's body to be assessed, and the appropriateness of the treatment and dosage of the therapeutic drug to be evaluated. The ability to monitor the distribution of therapeutic radioisotopes in real time enables the understanding of specific patient conditions and characteristics, and the selection of the most effective treatment through theranostics, facilitating personalized medicine (1–3).

The concept of using therapeutic and diagnostic radioisotopes in cancer treatment was first proposed in 1946 with the use of β^−^- and γ-emitting ^131^I (*T*_1/2_ = 8.0 d) for the treatment of thyroid cancer. Currently, 3.7–5.55 GBq of ^131^I is used for thyroid cancer treatment (3, 5). Common radioisotope pairs used for therapeutic and diagnostic purposes include ^131^I (^123^I, ^124^I), ^90^Y (^111^In, ^86^Y), ^177^Lu (^111^In), ^212^Pb (^68^Ga, ^86^Y), ^223^Ra (^99m^Tc, ^18^F), ^225^Ac (^68^Ga, ^86^Y), ^227^Th (^89^Zr), ^186^Re (^99m^Tc), and ^67^Cu (^64^Cu) (6). Diagnostic radioisotopes are indicated in parentheses.

**Figure 1:**
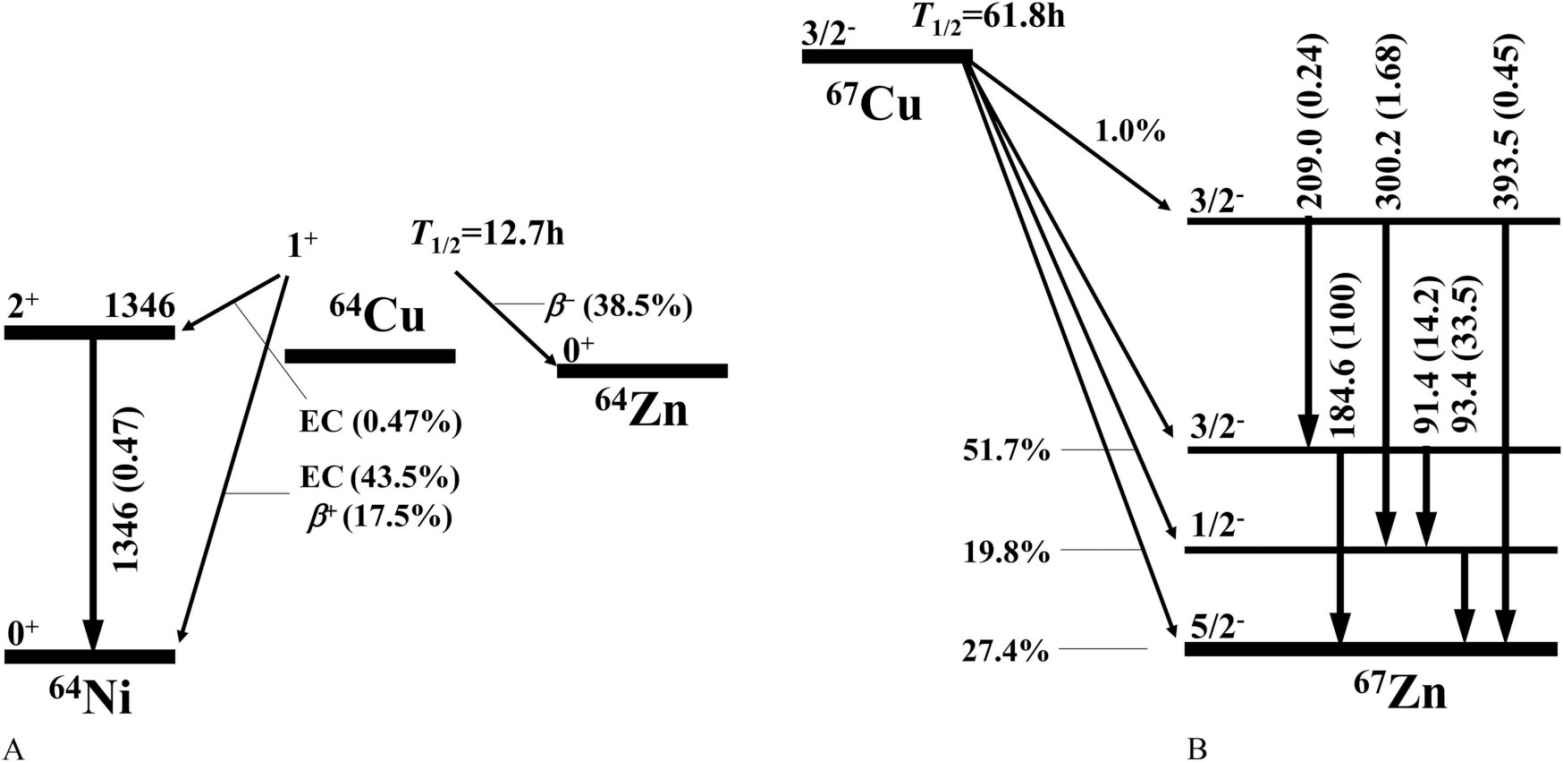
Decay schemes of ^64^Cu **(A)** and ^67^Cu **(B)**, reproduced from references (12) and (14), respectively.

Concurrently, the ^64^Cu/^67^Cu pair is an emerging set of radionuclides that are ideal for use in therapeutic diagnostics (theranostics) (7–11) due to their identical chemical and biological properties, the ability of copper to form diverse coordination complexes with small molecules, antibodies, and proteins, and the favorable physical properties of ⁶⁴Cu and ⁶⁷Cu (4–6). Specifically, ⁶⁴Cu has a half-life of 12.7 h and decays via positron emission (17.5%) with a maximum energy of 0.653 MeV, β^−^-rays emission (38.5%) with a maximum energy of 0.579 MeV, and electron capture (44.0%) (12, 13) as shown in Figure 1A, making it suitable for positron emission tomography (PET). ⁶⁷Cu has a half-life of 61.8 h and emits β^−^-rays with maximum energies of 0.377 MeV (57%), 0.468 MeV (22%), and 0.562 MeV (20%) (13, 14), as illustrated in Figure 1B. These β^−^-rays have a range of approximately 3 mm in water (15). Furthermore, ⁶⁷Cu emits 91, 93, and 185 keV γ-rays, enabling its detection by gamma cameras. Consequently, ^67^Cu is well-suited for diagnostic imaging and internal radiotherapy. Therefore, increasing Cu availability is crucial for the development of radiopharmaceuticals that target various diseases (6–8).

Numerous studies have been on the production of ⁶⁷Cu using the ⁶⁷Zn(n,p)⁶⁷Cu reaction in reactors (16). Additionally, studies have been conducted on the production of ⁶⁷Cu using the ⁶⁸Zn(p,2p)⁶⁷Cu (16, 17), ⁶⁸Zn(γ,p)⁶⁷Cu (18, 19), ⁶⁷Zn(n,p)⁶⁷Cu (20), ⁷⁰Zn(p,α)⁶⁷Cu (16, 21), and ⁷⁰Zn(d,αn)⁶⁷Cu (22) reactions in accelerators. Recently, a significant improvement in the accessibility of ⁶⁷Cu was achieved using the ⁶⁸Zn(γ,p)⁶⁷Cu reaction at the Argonne National Laboratory Low Energy Accelerator Facility (9). This resulted in a production yield of over 62.9 GBq after 53.5 h of irradiation.

^64^Cu has also been produced in reactors by the ^63^Cu(n,γ)^64^Cu reaction and in accelerators by the ^64^Ni(p,n)^64^Cu, ^64^Ni(d,2n)^64^Cu, ^64^Zn(d,2p)^64^Cu, ^66^Zn(d,*α*)^64^Cu, ^68^Zn(p,*α*n)^64^Cu, and ^64^Zn(n,p)^64^Cu reactions. The most commonly adopted production route is ^64^Ni(p,n)^64^Cu (23). Indeed, the production of high-quality ^64^Cu with 8.7 GBq at EOI was achieved by bombarding a highly enriched ^64^Ni target (enrichment 99.53%) with a 20 μA proton beam for a period of 4 h (24).

Kin et al. previously proposed a novel route for producing ^67^Cu and ^64^Cu using accelerator neutrons with energies ranging from a few MeV to approximately 40 MeV via the ^68^Zn(n,n'p)^67^Cu and ^68^Zn(n,d)^67^Cu reactions, as well as the ^64^Zn(n,p)^64^Cu reaction (15), based on the following results obtained by them: Kin et al. measured the activation cross-sections of ^64^Cu and ^67^Cu by bombarding natural zinc with 14 MeV neutrons. The production yields of ^64^Cu and ^67^Cu by accelerator neutrons from ^nat^C(d,n) with 40 MeV 5 mA deuterons were estimated using the results and the evaluated cross-sections of the Zn isotopes. The estimated ^64^Cu yield was 1.8 TBq (175 g ^64^Zn) after 12 h of irradiation. At the end of the two-day irradiation period, the estimated yield of ^67^Cu from ^67^Zn(n,p)^67^Cu was 249 GBq (184 g ^67^Zn), and the estimated yield from ^68^Zn(n,n'p)^67^Cu and ^68^Zn(n,d)^67^Cu were 287 GBq (186 g ^68^Zn).

Subsequently, production yield studies of ^67^Cu and ^64^Cu were conducted (25–28), and an apparatus for the separation and purification of these radionuclides from neutron-irradiated ZnO was constructed. Kawabata et al. initially established a fundamental separation and purification procedure, using only the column separation technique for ^64^Cu and ^67^Cu from neutron-irradiated ^nat^ZnO and ^64^ZnO 5 g with high separation efficiency and successful labelling, together with high recovery of Zn samples. The column separation technique was employed in the determination of the biodistribution of ^67^CuCl_2_ in colorectal tumor-bearing mice by Sugo, Hashimoto, Kawabata et al. (26). In the latest study, Kawabata et al. developed a combined thermal and column chromatography separation to separate ^64^Cu and ^67^Cu from 55.4 g of natural zinc that had been irradiated with accelerator neutrons. Sublimation removed 99% of the zinc, with 97% physically recovered for reuse. Following the removal of most of the zinc by thermal separation, the residual zinc containing ^67^Cu was in the order of milligrams. The residual zinc was further purified using chromatographic resins (28, 29). The experiments yielded a total separation efficiency of 73% for ^67^Cu.

In this study, we measured the absolute activity and radionuclidic purity of ^64^Cu and ^67^Cu produced by neutron irradiation of ^64^ZnO and ^68^ZnO. The measured values of ^64^Cu and ^67^Cu were compared with the simulation results. In addition, new sublimation and column separation apparatuses were installed at the Research Center for Accelerator and Radioisotope Science (RARiS) facility at Tohoku University.

## 2 Materials and methods

**Figure 2:**
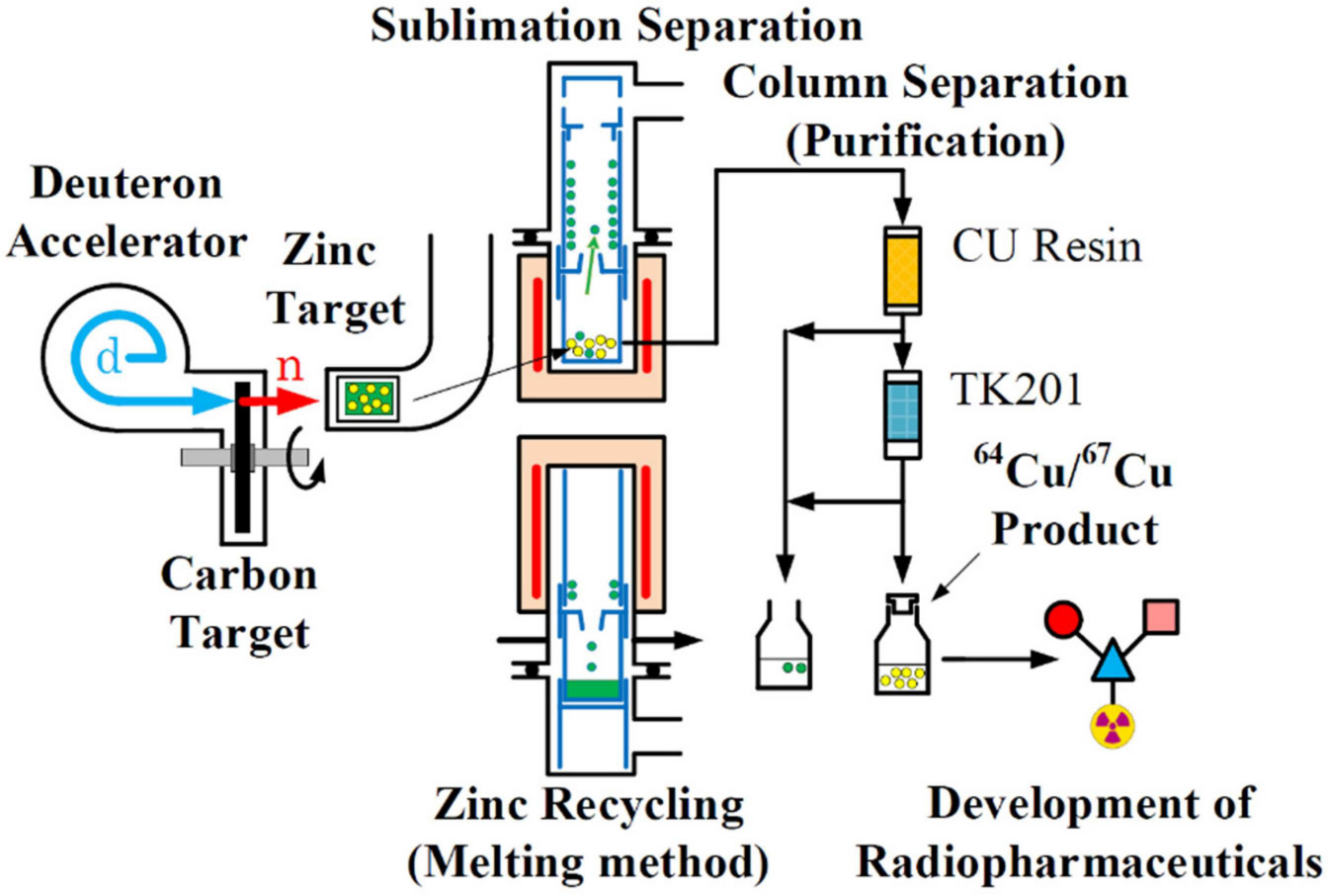
Schematic diagram of ^67^Cu and ^64^Cu production using accelerator neutrons generated by deuterons, and their subsequent separation from ^68^Zn and ^64^Zn. The accelerator neutrons were produced via the ^nat^C(d,n) reaction in a vacuum. The ^nat^C target was placed in a vacuum chamber and equipped with a rotating cooling system to manage the thermal load associated with high-intensity deuteron beams.

The schematic diagram for the production and separation of ^64^Cu and ^67^Cu from neutron-irradiated ^64^Zn and ^68^Zn is shown in Figure 2.

### 2.1 Production of ^67^Cu and ^64^Cu

Copper−64 and Copper-67 were produced by irradiating enriched samples of ⁶⁴Zn and ⁶⁸Zn with accelerator neutrons through the reactions ⁶⁴Zn(n,p)⁶⁴Cu, ⁶⁸Zn(n,n’p)⁶⁷Cu, and ⁶⁸Zn(n,d)⁶⁷Cu. Accelerator neutrons were generated via the ^nat^C(d,n) reaction. The natural carbon target (^nat^C), with a thickness of 10 mm and a diameter of 27 mm, was placed within a deuteron beam duct (vacuum). The samples, consisting of 0.295 g ^64^ZnO and 0.363 g ^68^ZnO, each with a diameter of 10 mm, were placed in air at 0° with respect to the deuteron beam direction. Prior to irradiation, the samples were pressed into pellets and sintered at 150°C for 40 min. The isotopic composition of the ^64^Zn sample was 99.935% and that of the ^68^Zn sample was: ^64^Zn (0.03 at%), ^66^Zn (0.16 at%), ^67^Zn (0.62 at%), ^68^Zn (99.16 at%), and ^70^Zn (0.03 at%). Niobium foils, 10 mm in diameter and 0.1 mm thick, were positioned on either side of the ^64^ZnO and ^68^ZnO pellets to monitor the neutron yield. A typical description of the experimental setup for irradiation of Zn samples is given in (28).

Copper-64 was produced by irradiating ^64^ZnO with neutrons for three hours. Neutrons were generated using 41 MeV deuterons with an average beam current of 0.11 μA, provided by the AVF cyclotron at the Takasaki Ion Accelerator Advanced Radiation Applications Facility (TIARA), National Institutes for Quantum and Radiological Science and Technology (30). Copper-67 was produced by irradiating ^68^ZnO with neutrons for three minutes. Neutrons were generated using 52 MeV deuterons with an average beam current of 0.379 μA, provided by the AVF cyclotron at RARiS, Tohoku University (31). The 52 MeV deuteron beam is the highest energy currently achievable with these AVF cyclotrons.

**Figure 3:**
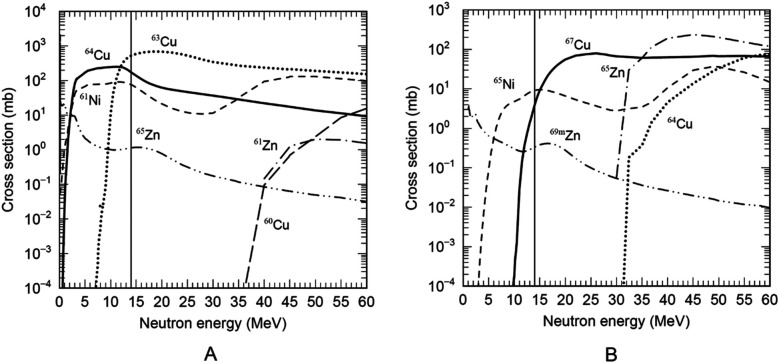
**(A)** evaluated excitation functions for reactions such as ^64^Zn(n,p)^64^Cu, ^64^Zn(n,n’p)^63^Cu, and ^64^Zn(n,d)^63^Cu during neutron irradiation of enriched ^64^Zn (32). **(B)** Evaluated excitation functions for reactions including ^68^Zn(n,n′p)^67^Cu, ^68^Zn(n,d)^67^Cu, and ^68^Zn(n,4n)^65^Zn during neutron irradiation of enriched ^68^Zn (32). ^65^Ni (T_1/2_ = 2.5 h) and ^65^Zn (T_1/2_ = 244 d) decay to ^65^Cu (a stable nuclide), while ^69m^Zn (T_1/2_ = 14 h) decays to ^69^Ga (also stable). The excitation functions for the ^68^Zn(n,n′p)^67^Cu, ^68^Zn(n,d)^67^Cu, and ^64^Zn(n,p)^64^Cu reactions indicate very low production of Zn-based impurity radionuclides. The most probable 14 MeV neutron energy generated by the ^nat^C(d,n) reaction using 40 MeV deuterons is indicated by the thin solid line (33).

The absolute activity and radionuclide purity of ⁶⁴Cu and ⁶⁷Cu were, respectively, determined by measuring the 1,346 keV γ-ray intensity from the decay of ⁶⁴Cu and the 185 keV γ-ray intensity from the decay of ⁶^7^Cu. The absolute activity of ⁶⁴Cu and ⁶⁷Cu depends on the excitation function for the reaction of ⁶⁴Zn(n,p)⁶⁴Cu and ⁶⁸Zn(n,n’p)⁶⁷Cu and the ⁶⁸Zn(n,d)⁶⁷Cu. The excitation functions evaluated for reactions such as ^64^Zn(n,p)^64^Cu, ^64^Zn(n,n’p)^63^Cu, and ^64^Zn(n,d)^63^Cu are shown in Figure 3A, and those evaluated for reactions such as ^68^Zn(n,n'p)^67^Cu, ^68^Zn(n,d)^67^Cu, and ^68^Zn(n,4n)^65^Zn are shown in Figure 3B. These figures highlight the importance of measuring the yields and radionuclide purities of ^64^Cu and ^67^Cu over a range of neutron (or deuteron) energies.

The accelerator neutrons used to produce ^64^Cu and ^67^Cu exhibited the following characteristics. First, a neutron energy spectrum can be obtained from the C(d,n) reaction, which is suitable for the efficient production of these radionuclides, by appropriately selecting the deuteron energy (*E*_in_) (27, 28). Second, neutrons are predominantly emitted in the forward direction relative to the deuteron beam axis (34–36), resulting in nearly complete irradiation of the enriched ^64^Cu or ^68^Zn placed immediately behind the ^nat^C target (36). Notably, SPIRAL2 at GANIL in France will produce 10^1^⁵ neutrons per second (n/s) through the ^nat^C(d,n) reaction using 40 MeV, 5 mA deuterons (33).

#### 2.1.1 Absolute activity and radionuclide purity of ^64^Cu and ^67^Cu

The measured absolute activities and radionuclide purities of ^64^Cu and ^67^Cu were compared with the evaluated values, as follows:

First, we note that a single radionuclide, B, is produced via a neutron-induced reaction on the Zn isotope A in the enriched ^64^Zn or ^68^Zn samples. This is represented by the A(n,x)B reaction. Next, the yield rate *Y_a_* of radionuclide B produced from isotope A via a reaction channel, *α* ≡ *α*(A, B), of the A(n,x)B reaction was derived as:

(1) $$\begin{array}{c} Y_{\alpha }=\overset{E_{\max}}{\underset{E_{\min}}{\int }}\;\sigma_{\alpha }(E_{n})f_{n}(E_{n})dE_{n}, \end{array}$$

where *σ_α_*(*E_n_*) is the excitation function at neutron energy *E_n_* for the channel *α*, and *f_n_*(*E_n_*) is the neutron fluence in the sample. In Equation 1, the limits *E*_min_ and *E*_max_ correspond to the energy range of the neutrons produced by the ^nat^C(d,n) reaction in a 10-mm-thick carbon target. Note that *E*_min_ should be set to the threshold energy $E_{\mathrm{th}-\alpha }$ for channel *α* if *E*_min_ is lower than this threshold. Excitation functions *σ_α_*(*E_n_*) were obtained from the production cross sections provided in the fifth version of the Japanese Evaluated Nuclear Data Library (JENDL-5) (37). The neutron fluence *f_n_*(*E_n_*) was derived from a particle transport simulation using the Particle and Heavy Ion Transport code System (PHITS) (38), which accounted for neutron propagation from the carbon target—where neutrons are produced with energy $E_{n}^{\prime }$ and angle $\Omega_{n}^{\prime }$ *via* the ^nat^C(d,n) reaction—to the Zn sample. The fluence of the produced neutrons at position ***r*** in the target is given by:

(2) $$\begin{array}{c} {\bar{\;f}}_{n}(E_{n}^{\prime },\Omega_{n}^{\prime },r)=N_{C}\overset{E_{\mathrm{in}}}{\underset{0}{\int }}\;\sigma_{\left(d,n\right)}(E_{d},E_{n}^{\prime },\Omega_{n}^{\prime })f_{d}(E_{d},r)dE_{d}, \end{array}$$

where $N_{C}$ is the number of carbon nuclei in the target, $E_{\mathrm{in}}=41$ and 52 MeV are the incident deuteron energies, $\sigma_{\left(d,n\right)}$ are the neutron production cross sections obtained from JENDL-5, and $f_{d}$ is the deuteron fluence at position ***r***, normalized per incident deuteron. The PHITS simulation accounted for the attenuation of deuteron fluence in the target, including the corresponding decrease in deuteron energy. The neutron fluence $f_{n}$ in the Zn sample was calculated as the component of ${\bar{\;f}}_{n}$ directed toward the sample. By setting the number of deuterons *N_d_* irradiating the sample, isotopic abundance *R_A_* of isotope A in the Zn sample, and particle density *ρ* of Zn, the total yield *Y*(B) of radionuclide B in the sample can be expressed as:

(3) $$\begin{array}{c} Y(B)=N_{d}\underset{A}{\sum }\;R_{A}\rho \underset{\alpha \in B}{\sum }\;Y_{\alpha }, \end{array}$$

where α∈B indicates that the summation includes all reaction channels *α* through which isotope A can produce radionuclide B. Here, A refers to one of the five stable Zn isotopes: ^64^Zn, ^66^Zn, ^67^Zn, ^68^Zn, and ^70^Zn, and B denotes one of Cu or Zn radionuclides listed in Reference (39). Furthermore, Ga radionuclides were evaluated as products B by replacing neutrons with protons in the above equations. Finally, the yield was multiplied by the saturation factor to evaluate the amount produced at the EOI. Stable isotopes of ^63^Cu and ^65^Cu can be produced by reactions such as ^64^Zn(n,n'p)^63^Cu and ^68^Zn(n,p3n)^65^Cu, respectively. Their production reduced the specific activity of the products ^64^Cu and ^67^Cu. Consequently, an absolute yield evaluation of these isotopes is imperative, because they are not detectable by radiation detectors.

### 2.2 Separation of ^67^Cu and ^64^Zn from irradiated ^68^Zn and ^nat^Zn

We developed a new sublimation and column chromatography separation apparatus to be installed in an existing hot cell at the Research Center for Accelerator and Radioisotope Science (RARiS) facility at Tohoku University. The apparatus was designed for the production and separation/purification of ^64^Cu and ^67^Cu using the cyclotron at Tohoku University. To obtain ^67^Cu and ^64^Cu from irradiated ^68^Zn and ^nat^Zn, respectively, the same separation apparatus was used for both sublimation and column chromatography because both irradiated samples contained common impurity radionuclides belonging to Cu and Zn in addition to the desired ^67^Cu and ^64^Cu.

#### 2.2.1 Sublimation separation of Zn

**Figure 4:**
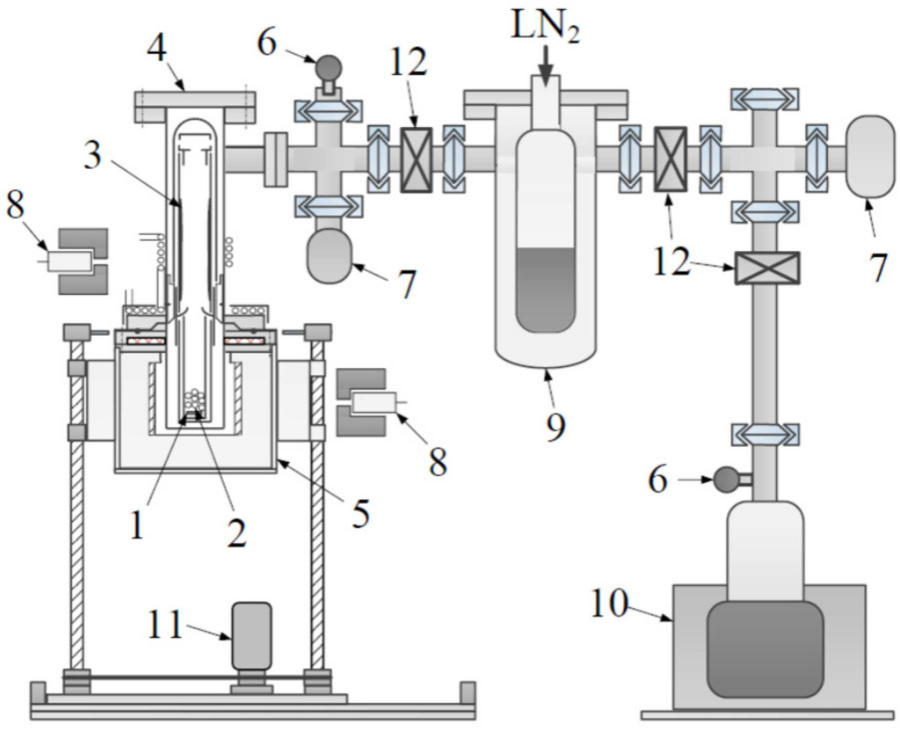
Layout of the sublimation separation apparatus for an irradiated Zn sample. (1) Irradiated Zn sample containing ^67^Cu activity, (2) quartz beads, (3) sublimated Zn element, (4) vacuum chamber, (5) electric tubular furnace, (6) electric leak valve, (7) vacuum gauge, (8) CZT *γ*-ray detector, (9) cold trap, (10) turbomolecular pump, (11) motor, (12) butterfly valve.

The initial separation of ^67^Cu (or ^64^Cu) in the milligram range from neutron-irradiated bulk ^68^Zn (or ^nat^Zn) was achieved using the sublimation method, originally developed by the Argonne National Laboratory (ANL) group (40). We developed a vertical-type sublimation apparatus (Figure 4) instead of the horizontal-type due to the inadequate effective dimensions within the cell.

Cooling devices were attached to the upper flange and top of the vacuum vessel to facilitate the recovery of sublimated Zn inside the system. Sublimation is an effective method for the separation of Zn and Cu due to the substantial difference in their boiling points: The boiling points of Zn and Cu are 907°C and 2,562°C, respectively. In this study, sublimation experiments were performed at temperatures of 500 and 600°C under a vacuum of 2.0 × 10^−5^ hectopascal (hPa) to determine the optimal temperature for maximizing Zn sublimation while reducing the amount of distilled Cu. This apparatus can separate more than 40 g of Zn via thermal separation.

The furnace temperature can be controlled remotely, and the separation vessel can be raised or lowered remotely outside the cell. Two ^67^Cu activities were produced by irradiating 43.6 g of ^nat^Zn, consisting of three pellets, with accelerator neutrons, and bombarding an enriched ^68^Zn pellet (3.93 g) with photons. Accelerator neutrons and photons were generated by a cyclotron and an electron linear accelerator (linac), respectively, at Tohoku University. The irradiated Zn samples were placed in a quartz tube with quartz beads (7 mm ϕ), which were dispersed between the pellets to serve as fillers and increase the sublimation surface among samples comprising more than 10 g of multiple pellets to increase the sublimation surface area of the Zn and shorten the sublimation time (28). γ-ray spectra were recorded every 3 min using a CZT detector while heating, in order to monitor changes in the activity of ^67^Cu (185 keV) and ^65^Zn (1,116 keV) over time. After heating was stopped, the furnace was lowered to allow the system to cool below 200°C, after which the equipment was returned to atmospheric pressure. The quartz components were then removed and weighed to determine the Zn deposition and sublimation efficiency.

The efficiency of sublimation separation of irradiated Zn was determined online by comparing the activity of ⁶⁵Zn (1,116 keV γ-ray) measured with the CZT detector before and after separation. The Zn accumulated in the quartz tube during the sublimation of Cu from the irradiated Zn was collected for recycling. Prior to collection, the distribution of accumulated Zn was determined by measuring the ^65^Zn (1,116 keV) signal using a high-purity germanium (HPGe) detector. The separation yield of Zn was calculated using a gravimetric method.

The non-radioactive Cu present in the Zn sample was removed prior to irradiation, because it lowers the specific activity of the ^64^Cu or ^67^Cu product (28). The zinc samples were sublimated at 650°C for 120 min under vacuum to prevent oxidation. The sublimated zinc was subsequently collected, melted, and prepared as pellets for irradiation. This method can also be applied to the recycling of enriched zinc after the separation of ^67^Cu (^64^Cu).

The copper remaining in the test tube was further purified using commercially available resins, as described below.

#### 2.2.2 Purification of ^67^Cu by column chromatography

**Figure 5:**
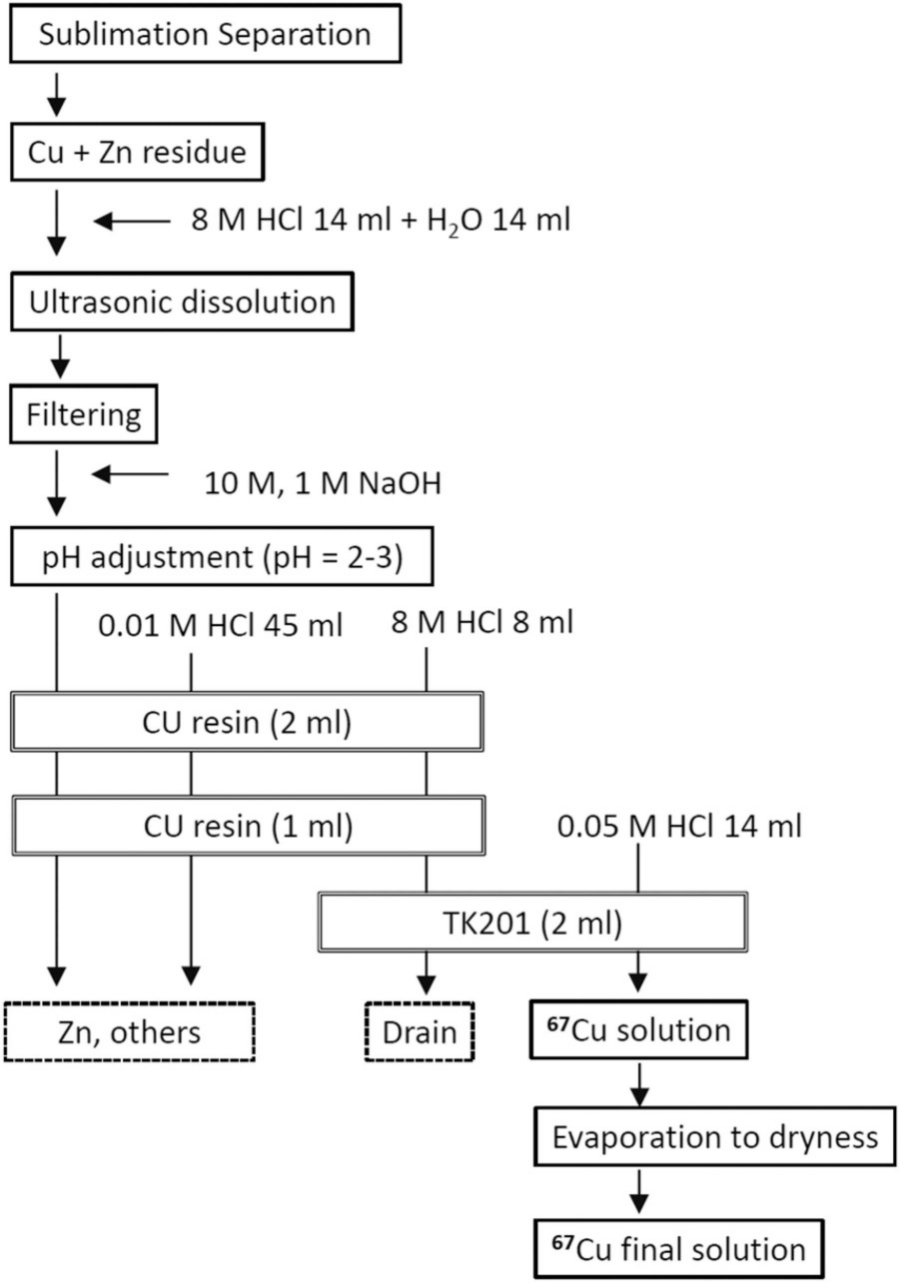
Purification steps of ^67^Cu from Zn.

The purification scheme is illustrated in Figure 5. Two CU Resin cartridges (2 ml and 1 ml, TrisKem International) and one TK201 cartridge (2 ml, TrisKem International) were pre-conditioned with 30 ml of 0.01 M HCl (15 ml for the 1 ml resin) and 30 ml of 8 M hydrochloric acid, respectively. A 1 ml CU Resin cartridge was placed below the 2 ml cartridge as a guard column to retain any copper that may have leaked from the larger cartridge. After the sublimation of bulk Zn, quartz beads were added to the test tube to fill the void space, and 8 M hydrochloric acid was added until the tube opening was submerged, —typically requiring approximately 15 ml, —to ensure the complete dissolution of the copper and Zn residues. Dissolution was enhanced by applying ultrasonic waves for 10 min. The solution containing dissolved Cu and Zn was transferred to another container. The test tubes were washed with ultrapure water under ultrasonic agitation for 10 min. The resulting solution was filtered through a glass filter to remove insoluble residues and ash. The pH of the filtrate was adjusted to pH 2–3 using a NaOH solution.

This pH-adjusted solution was loaded onto the CU Resin cartridge to adsorb ^67^Cu, followed by washing with 45 ml of 0.01 M HCl to remove residual Zn and other impurities. The flow rate was maintained at 1.0 ml/min, controlled by a peristaltic pump. After trapping copper in the CU Resin, 8 ml of 8 M HCl was passed through the column to elute ^67^Cu, which was then reabsorbed onto the TK201 resin. The final ^67^Cu product was eluted with 14 ml of 0.05 M HCl, followed by acid removal through evaporation. The same purification procedure was use to separate ^64^Cu from ^64^Zn.

The total time required for sublimation and chromatography steps was 8 h, each step requiring 4 h.

## 3 Results

We discuss these results by referring to the *γ*-ray branching ratios obtained from the decay of ^67^Cu, as shown in Figure 1A.

### 3.1 Absolute activity and radionuclide purity of ^64^Cu and ^67^Cu

**Figure 6:**
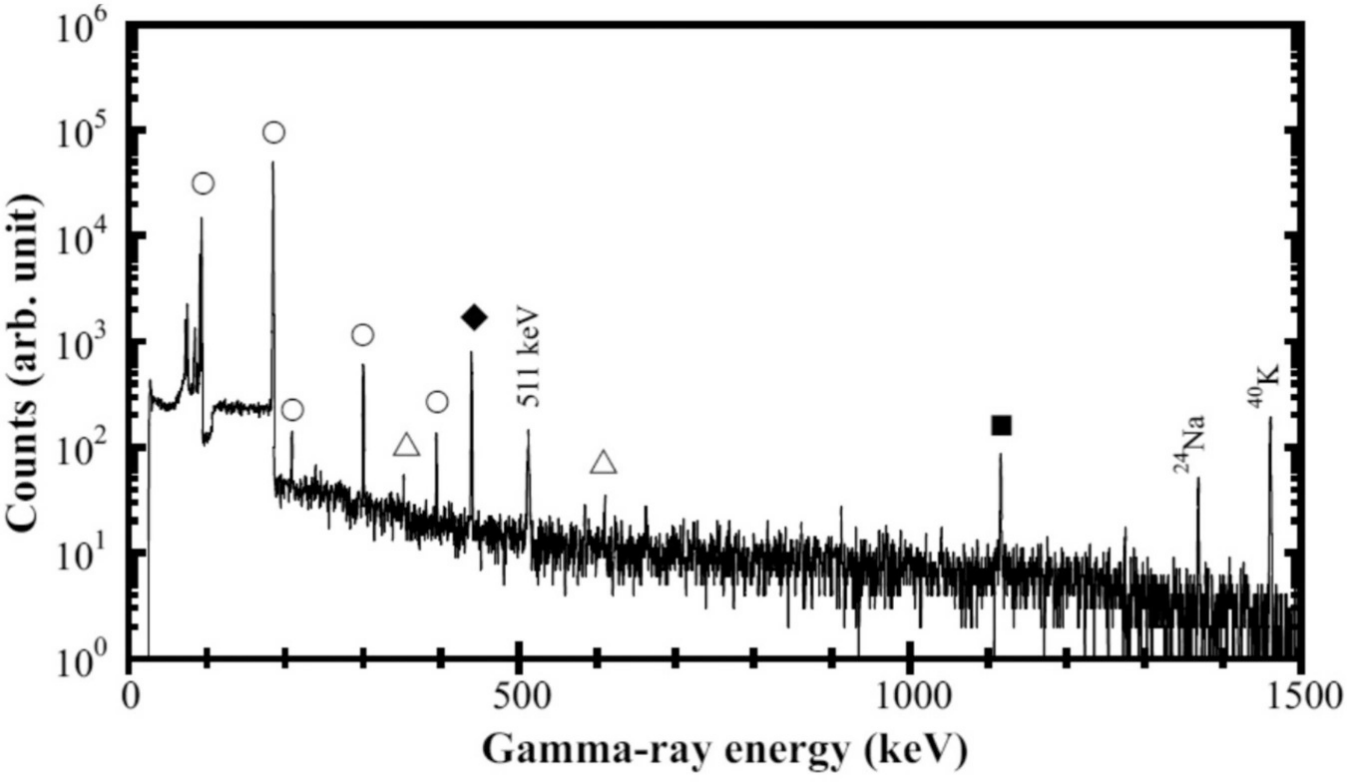
γ-ray spectrum of the neutron irradiated ^68^ZnO using a deuteron beam of 52 MeV. The γ-ray peaks originate from the decays of ^67^Cu (open circles), ^65^Ni (open triangles), ^69m^Zn (filled diamond), and ^65^Zn (filled square).

Figure 6 shows the *γ*-ray spectrum of irradiated ^68^Zn. The observed γ-ray peaks originate from the decay of ^67^Cu (91, 93, 185, 209, 300, and 394 keV), ^65^Ni (T_1/2_ = 2.52 h; 366, 508, 610, and 1,116 keV), ^65^Zn (T_1/2_ = 244 d; 1,116 keV), and ^69m^Zn (T_1/2_ = 13.8 h; 439 keV). The isotope assignments of the observed *γ*-rays were based on their energies, decay curves, and known branching ratios.

The activity of ^67^Cu and the impurity of ^65^Zn (as well as ^64^Cu and the impurities of ^61^Cu, ^62^Zn, and ^63^Zn) at EOI were determined by considering the branching ratios of the observed 185 keV and 1,116 keV (and 1,346 keV and 656 keV, 597 keV, and 670 keV) *γ*-rays (41), as well as the γ-ray detection efficiency of the HPGe detector, which was calibrated using a standard ^152^Eu γ-ray source. The self-absorption of the *γ*-rays in the irradiated ^68^Zn sample was corrected using the photon cross-sectional database provided by the National Institute of Standards and Technology (42). The calculated yields of ^67^Cu and ^65^Zn (along with ^64^Cu, ^61^Cu, ^62^Zn, and ^63^Zn) at EOI, obtained using the radionuclide production rates, irradiation time, and deuteron beam intensity, are presented in Table 1. The calculated and measured yields were in good agreement within an uncertainty of ±20%. The total systematic uncertainty in the calculated yields was estimated to be 23%, taking into account the uncertainty of 18% in the measured neutron data for the ^nat^C(d,n) reaction (43), and an assumed 15% uncertainty in the evaluated cross sections. The total systematic uncertainty in the experimental values was calculated to be 12%, based on the estimated uncertainties in the distance between the carbon target and the sample, spatial distribution of the deuteron beam intensity and diameter, and *γ*-ray detection efficiency of the HPGe detector.

**Table 1:** The measured and calculated activities of ^67^Cu, ^64^Cu, ^65^Zn, ^69m^Zn, ^65^Ni, ^66^Ga, and ^67^Ga at the EOI of enriched ^68^Zn at *E*_in_ = 52 and 40 MeV are shown.

Radionuclide	^67^Cu	^64^Cu	^65^Zn	^69m^Zn	^65^Ni	^66^Ga	^67^Ga	^65^Cu/^67^Cu	^63^Cu/^67^Cu
T1/2	2.58 d	0.53 d	244 d	0.57 d	0.11 d	0.39 d	3.2 d		
Unit	Bq	Bq	Bq	Bq	Bq	Bq	Bq	Atoms/cc	Atoms/cc
52 MeV									
Exp.	1.23 × 10^3^	<5 × 10^−1^	2.9	8.7 × 10^1^	ND	ND	ND		
Cal.	1.20 × 10^3^	8.1 × 10^1^	4.3	1.1 × 10^2^	5.5 × 10^3^	1.1 × 10^−1^	7.2 × 10^−1^	0.13	0.004
40 MeV									
Exp.	3.83 × 10^2^	<6	2.5 × 10^−1^	5.3 × 10	ND	ND	ND		
Cal.	3.88 × 10^2^	7.5	4.5 × 10^−1^	5.7 × 10	2.4 × 10^3^	5.1 × 10^−3^	8.4 × 10^−2^	0.05	0.005

The table also presents the ratio of calculated numbers of atoms for non-radioactive ^65^Cu and ^63^Cu relative to ^67^Cu. ND = not detected.

The measured activity of ^67^Cu at *E*_in_ = 52 MeV at EOI was 1.23 ± 0.05 kBq, which was approximately 3.2 times higher than 0.383 ± 0.02 kBq measured at *E*_in_ = 40 MeV. In contrast, the measured yield of ^65^Zn at 52 MeV was 2.9 ± 0.16 Bq, which was approximately 12 times higher than 0.25 ± 0.02 Bq, measured at 40 MeV. The substantial increase in ^67^Cu yield observed when employing 52 MeV deuteron beams underscores the practical advantage of expanding the availability of ^67^Cu, ^65^Zn was separated from ^67^Cu via sublimation.

**Figure 7:**
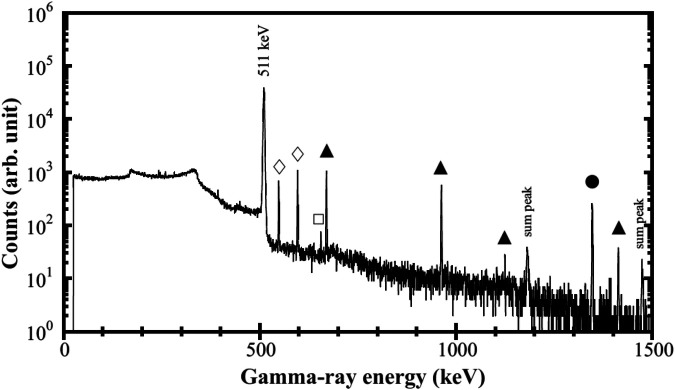
γ-ray spectrum of the neutron irradiated ^64^ZnO using a deuteron beam of 41 MeV. The γ-ray peaks come from the decays of ^64^Cu (filled circle), ^61^Cu (open square), ^62^Zn (filled tringle), and ^63^Zn (open diamond).

Figure 7 shows the gamma-ray spectrum of irradiated ^64^Zn. The observed gamma-ray peaks originate from the decay of ^64^Cu (511 and 1346 keV), ^61^Cu (T^1/2^ = 3.32 h, 656 keV), ^62^Zn (T^1/2^ = 9.26 h, 548.4 and 596.6 keV) ^63^Zn (T^1/2^ = 38.5 minutes. 669.6 and 962.1 keV). The measured activities of ^64^Cu, ^61^Cu, ^62^Zn, and ^63^Zn at *E*^in^ = 41 MeV at the EOI were 6.61  ± 0.42 kBq, 59.4 ± 4.7 Bq, 214 ± 12 Bq, and 39.5 ± 3.6 kBq, respectively, as listed in Table 2. ^64^Cu produced the low amount level of ^61^Cu radioactive waste. Copper-61 is produced by the ^64^Zn(n,p3n)^61^Cu reaction. Short lived impurity radionuclide ^62^Zn and ^63^Zn can be separated by sublimation process.

**Table 2:** The measured and calculated activities of ^64^Cu, ^61^Cu, ^62^Zn, ^63^Zn, and ^65^Zn at the EOI of enriched ^64^Zn at *E*_in_ = 41 MeV are shown, along with the ratio of calculated numbers of atoms for non-radioactive ^63^Cu and ^65^Cu relative to ^64^Cu.

Radionuclide	^64^Cu	^61^Cu	^62^Zn	^63^Zn	^65^Zn	^63^Cu/^64^Cu
T1/2	0.53 d	3.33 h	9.26 h	38.5 m	244 d	
Unit	Bq	Bq	Bq	Bq	Bq	Atoms/cc
41 MeV						
Exp.	6.61 × 10^3^	5.94 × 10^1^	2.14E × 10^2^	3.95 × 10^4^	<2.0 × 10^−1^	
Cal.	7.04 × 10^3^	7.45 × 10^1^	1.62E × 10^2^	4.39 × 10^4^	1.9 × 10^−1^	3.8

ND, not detected.

### 3.2 Separation

**Figure 8:**
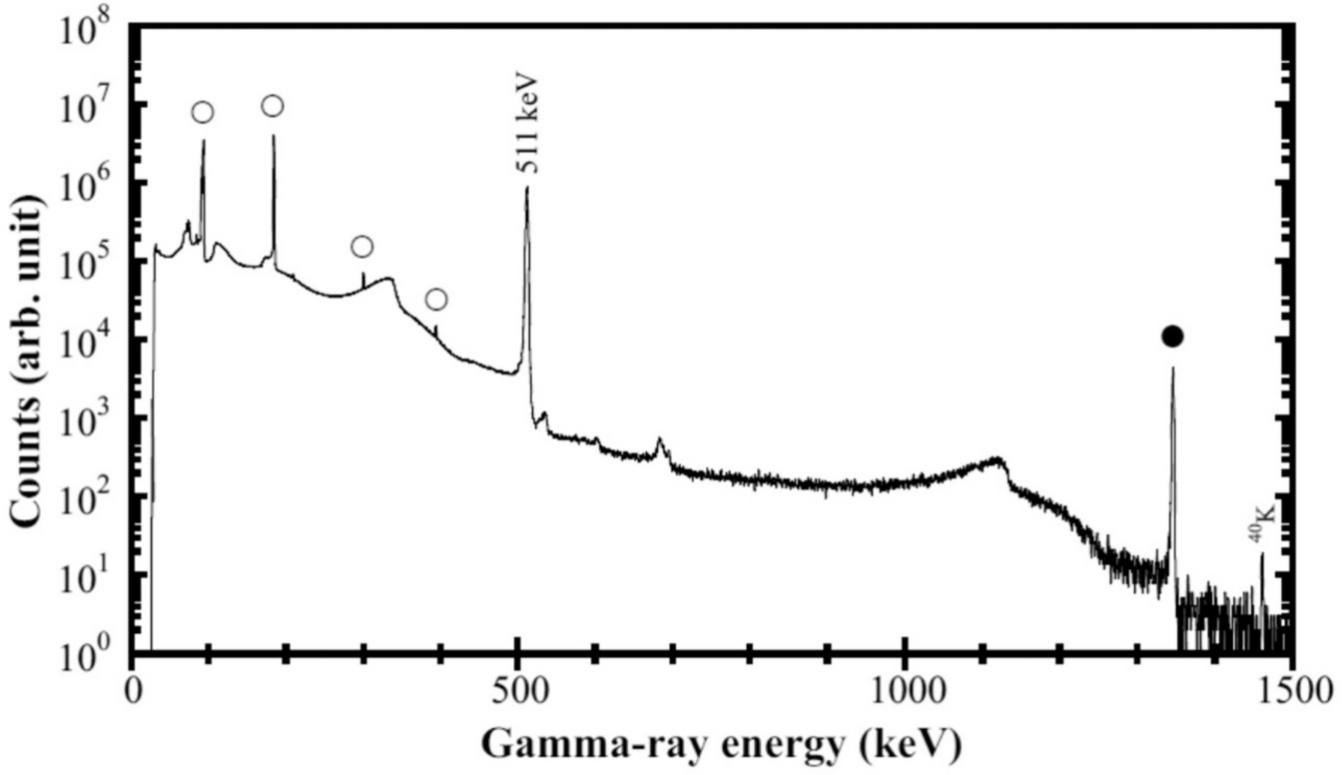
γ-ray spectrum measured after the purification of the neutron irradiated ^nat^ZnO using a deuteron beam of 40 MeV. The *γ*-ray peaks come from the decays of ^64^Cu (filled circle) and ^67^Cu (open circle).

Figure 8 shows the *γ*-ray spectrum measured after the purification of the neutron irradiated ^nat^ZnO. The *γ*-ray peaks come from the decays of ^64^Cu and ^67^Cu. A ^65^Zn radionuclide impurity in the final ^64^Cu product was below the detection limit of gamma-ray spectrometry providing ^65^Zn/^64^Cu\0.01%.

The Zn separation yield was determined using a gravimetric method. Using this apparatus, thermal separation was performed on 43.6 g of irradiated natural zinc, achieving a sublimation rate of 99% and recovering 94% of the zinc in a reusable form. At the end of the thermal separation process, the Cu-67 yield reached 87%, and subsequent chemical purification resulted in an overall Cu-67 recovery yield of 79%. For 3.932 g of enriched ^68^Zn, the separation efficiency was 99% by sublimation and 95% by column chromatography, with a total separation efficiency of 94%. The sublimation time for ^nat^Zn was 93 min, whereas that for ^68^Zn was 20 min. In both samples, the amount of ^67^Cu remaining in the non-sublimated material was less than 50 mg, which enabled subsequent purification by chromatography.

## 4 Discussion

The ^64^Cu/^67^Cu pair is an emerging set of radionuclides for use in theragnostic due to their identical chemical and biological properties and their favorable physical properties. Therefore, increasing the availability of ⁶⁷Cu and ⁶⁴Cu is crucial for developing radiopharmaceuticals that target various diseases. The novel production method of both ^67^Cu and ^64^Cu using accelerator neutrons provided from accelerators was previously proposed.

The absolute activity and radionuclidic purity of ^64^Cu and those of ^67^Cu were measured for the first time using enriched ^64^ZnO and ^68^ZnO at a deuteron energy of 41 MeV and 52 MeV, respectively. High radionuclidic purity of ^64^Cu was produced with a minimum level of radioactive waste. The ^67^Cu activity at 52 MeV was found to be 3.2 times higher than that at 40 MeV. The measured radioactivity and radionuclidic purity of ^64^Cu and ^67^Cu were in good agreement with the simulation based calculated values. The simulation further estimated the unmeasured yields of non-radioactive ^63^Cu and ^65^Cu relative to ^64^Cu and ^67^Cu at 41 MeV and 52 MeV, respectively. This information provides valuable insight into the specific activity of ^64^Cu and ^67^Cu.

Using the new apparatus, thermal separation was performed on 43.6 g of irradiated ^nat^Zn, achieving a sublimation efficiency (rate) of 99% and recovering 94% of the zinc in a reusable form. At the end of the thermal separation process, the ^67^Cu yield reached 87%, and subsequent chemical purification resulted in an overall ^67^Cu recovery yield of 79%. Separation experiments for ^64^Cu and ^67^Cu were conducted using neutron irradiated ^nat^Zn of 43.6 g and enriched ^68^Zn of 3.932 g. The sublimation temperature for the irradiated Zn was adjusted to 600°C and 500°C to enhance the Zn sublimation yield while minimizing the co-distillation of copper. Gamma-ray spectrum measured after the purification of the ^nat^ZnO show the dominant *γ*-ray peaks from the decays of ^64^Cu and ^67^Cu.

The present study demonstrates the fundamental steps for large-scale production of ^64^Cu and ^67^Cu. Deuteron beam intensity of 40 MeV used in this study was approximately 5 μA, 0.1% of 5 mA 40 MeV. As a result of this work, a new project aimed at accelerating 25–40 MeV, 100 µA (20 times the current intensity) deuterons using the existing cyclotron at RARiS, Tohoku University, has been approved (44). This capability is expected to be achieved in the near future.

## 5 Conclusion

The radionuclide pair ^64^Cu and ^67^Cu is considered an ideal theranostic candidate due to its identical chemical properties, the versatile coordination chemistry of copper, and suitable physical characteristics. Accordingly, radiopharmaceuticals based on ^64^Cu and ^67^Cu are expected to play a key role in the theranostic treatment of various diseases.

A novel method for producing ^64^Cu and ^67^Cu pairs using accelerator neutrons has demonstrated excellent results, enabling the separation and purification of high-quality ^64^Cu and ^67^Cu pairs using the same separation apparatus by sublimation and column chromatography-based separation of ^64^Cu and ^67^Cu from irradiated ^64^Zn and ^68^Zn. The central objective of the newly approved project is to strengthen ongoing research into the domestic production of radiopharmaceuticals containing ^64^Cu and ^67^Cu.

## Data Availability

The raw data supporting the conclusions of this article will be made available by the authors, without undue reservation.
